# Experimental observation of N00N state Bloch oscillations

**DOI:** 10.1038/ncomms9273

**Published:** 2015-09-22

**Authors:** Maxime Lebugle, Markus Gräfe, René Heilmann, Armando Perez-Leija, Stefan Nolte, Alexander Szameit

**Affiliations:** 1 Institute of Applied Physics, Abbe Center of Photonics, Friedrich-Schiller-Universität Jena, Max-Wien-Platz 1, 07743 Jena, Germany

## Abstract

Bloch oscillations of quantum particles manifest themselves as periodic spreading and relocalization of the associated wave functions when traversing lattice potentials subject to external gradient forces. Albeit this phenomenon is deeply rooted into the very foundations of quantum mechanics, all experimental observations so far have only contemplated dynamics of one and two particles initially prepared in separable local states. Evidently, a more general description of genuinely quantum Bloch oscillations will be achieved on excitation of a Bloch oscillator by nonlocal states. Here we report the observation of Bloch oscillations of two-particle N00N states, and discuss the nonlocality on the ground of Bell-like inequalities. The time evolution of two-photon N00N states in Bloch oscillators, whether symmetric, antisymmetric or partially symmetric, reveals transitions from particle antibunching to bunching. Consequently, the initial states can be tailored to produce spatial correlations akin to those of bosons, fermions and anyons, presenting potential applications in photonic quantum simulation.

## Abstract (web summary)

Bloch oscillations consist of periodic spreading and relocalization of particle wave functions, but have been so far observed only in separable states. Here the authors observe them for two-photon N00N states in integrated photonic circuits, revealing transitions from particle bunching to anitbunching.

## Graphical abstract

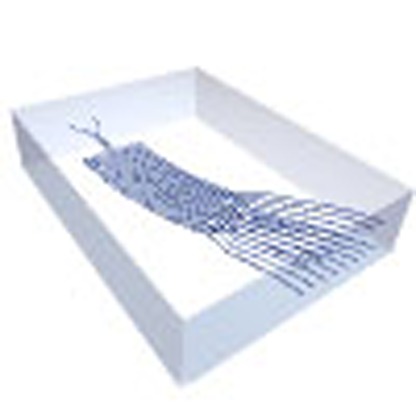

In his pioneering work^1^, Felix Bloch predicted the existence of periodic spatial oscillations of electrons traversing crystalline structures driven by linear external forces. At that time, understanding the concept of Bloch oscillations (BOs) contributed to the establishment of the electronic band structure theory in solids, which later on impacted wide areas of physics. In fact, BOs are a universal wave phenomenon and have since been formally investigated using diverse physical platforms^2^,^3^,^4^. Thus, while the observation of BOs is still elusive in its original conceived setting, a formal analogy between the propagation of classical beams and the evolution of point-like quantum particles can be appreciated. It has led to experimental realizations of photonic BOs in arrays of evanescently coupled waveguides^5^,^6^,^7^.

The aim of our work is to investigate BOs of two-particle N00N states and their correlations, on the basis of the theoretical work by Bromberg *et al*^8^. Our approach is based on integrated photonic circuits, a propitious platform to study fundamental quantum phenomena such as random walks^9^,^10^,^11^ as well as possible building blocks for quantum photonic chips^12^,^13^. The monolithic nature of such integrated devices gives access to full interferometric control over the field dynamics. As such, they are ideal candidates for the realization of quantum simulators^14^,^15^,^16^,^17^ capable to explore physical processes beyond the classical limits. Moreover, using photons makes it feasible to define N00N states on the spatial degree of freedom; since therein no particle–particle interaction can hinder the observation of interference effects.

In this article, we demonstrate that, as a result of spatial entanglement and quantum coherence, the amplitude interference occurring during the BOs of two-particle N00N states strongly influences the symmetry property of the propagating wave function^18^,^19^,^20^, giving rise to a bunching/antibunching cycle. Furthermore, we provide experimental evidence that the symmetry imposed on the initial state results in a spatial longitudinal shift of this cycle. In the context of quantum simulators^21^,^22^,^23^, this suggests a possibility for tailoring the bunching/antibunching behaviour of two-particle states, with spatial correlations observed along the cycle that are successively allusive to those exhibited by bosonic, anyonic and fermionic wave functions^24^.

## Results

### Theory of nonlocal Bloch oscillations

**Figure 1:**
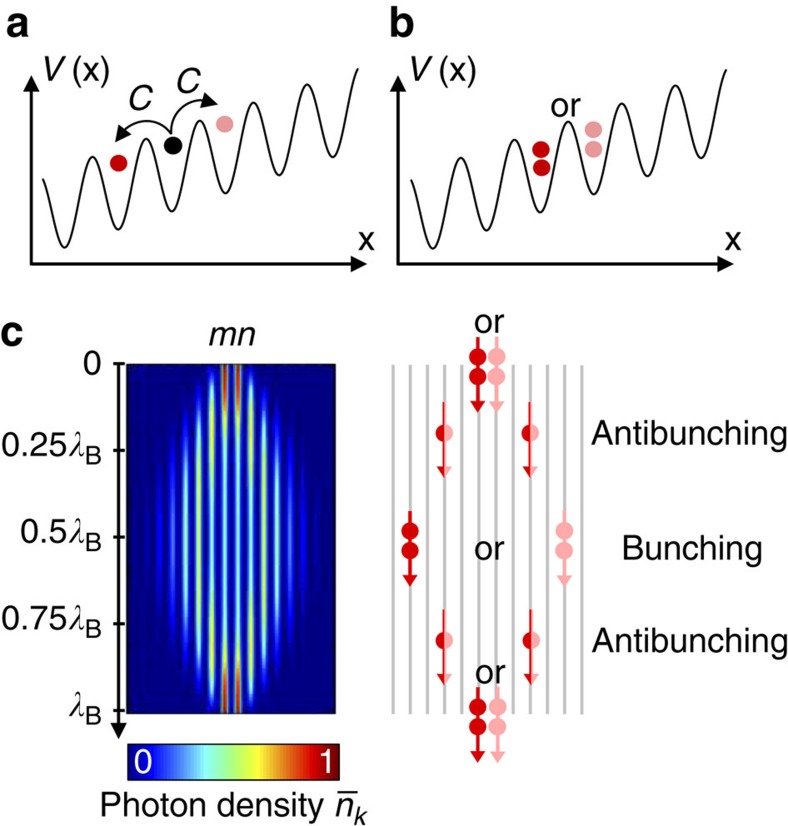
Quantum Bloch oscillator. (**a**) Bloch oscillations of a single particle when subject to a periodic potential *V*(x) with an external constant force acting on it, resulting in state entanglement (displayed as different colours). The coupling coefficient is denoted as *C*. (**b**) Bloch oscillations of the state 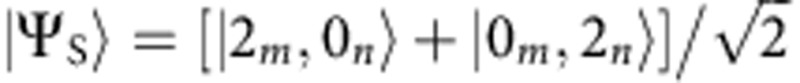 input in adjacent modes, implying probability interference that may either be constructive or destructive. (**c**) Response of a Bloch oscillator initially excited with the 
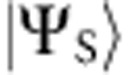
 state. The photon density 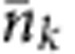 shows a perfect revival over one Bloch period *λ*_B_ (left inset). The corresponding correlation dynamics are schematically presented in the right inset. It conceptualizes the bunching to antibunching transitions governing the state all over the cycle.

To study the quantum dynamics of a tight-binding BO lattice^7^,^18^, we consider the Heisenberg equations of motion for field operators 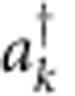 in the *k*th site


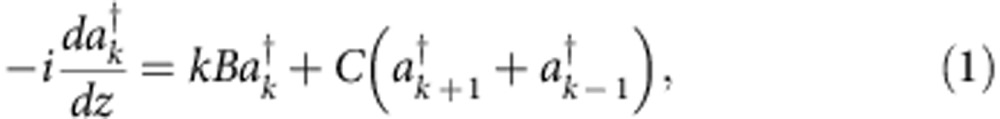


where *z* is the spatial coordinate mapping the time variable, *C* is the intermode coupling coefficient and *B* denotes the difference in the propagation constants of adjacent modes (Fig. 1a). The Bloch period *λ*_B_=2*π*/*B* is defined as the distance after which a revival of the state is expected. Integration of equation (1) yields the evolution of the creation operators 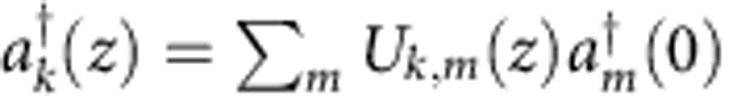 with 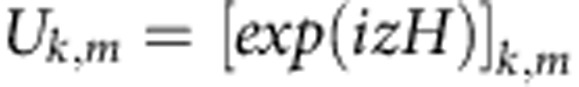 representing the evolution matrix, and *H* the coupling coefficient matrix.

In our work, we consider two-photon N00N states as the input of the BO lattice (Fig. 1b). Generally speaking, N00N states are maximally path-entangled multi-photon states describing an equal coherent superposition of all *N* particles being in one of two modes. Quantum features in this system have been theoretically addressed before our work and, most importantly, the spatial correlation function between multiple photons prepared in a N00N state was derived^8^. In the case of a two-photon N00N state coupled with adjacent lattice sites, the photon density 
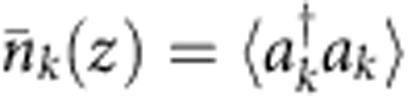
 shows a substantial broadening of the wave function up to half the Bloch cycle, further followed by a relocalization over *λ*_B_ (Fig. 1c, left inset). On the other hand, two-particle intensity correlations^18^, 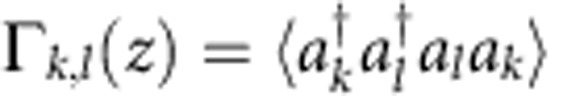, reveal that the presence of the ramping potential forces the photons to separate and gather cyclically with only half the Bloch period (Fig. 1c, right inset). In other words, bunching to antibunching transitions, and vice versa, is expected to occur at particular distances, which we will refer to as correlation turning points throughout our manuscript. In the general case of N-photon N00N states, the bunching to antibunching oscillation period was shown to be inversely proportional to *N* and to the initial separation between input modes^8^.

**Figure 2:**
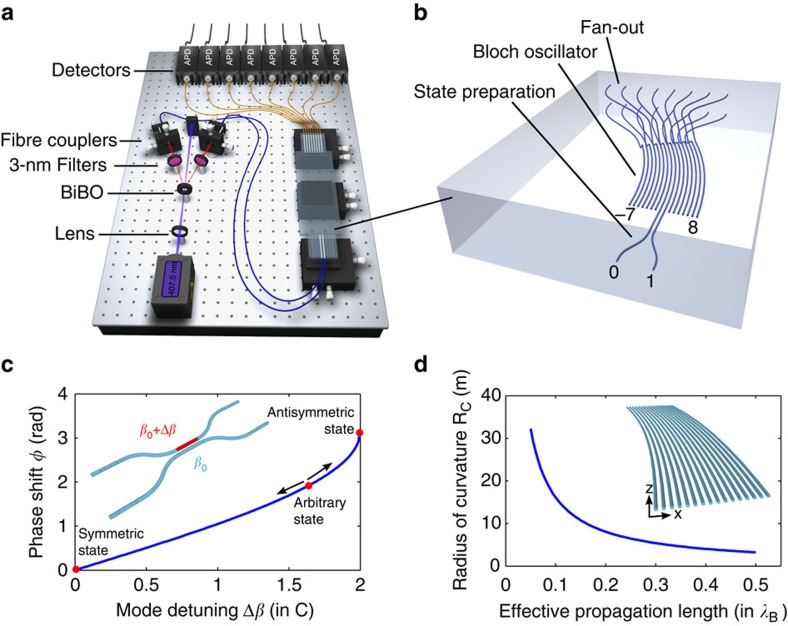
Experimental realization of Bloch oscillations of two-photon N00N states. (**a**) Spatial correlations measurement set-up. Photon pairs are produced by type-I spontaneous parametric down-conversion and single-photon detection is achieved using avalanche photodiodes. (**b**) Femtosecond-laser-written photonic circuit made of two functional sections: A two-photon N00N state with arbitrary symmetry is first prepared in a detuned directional coupler, and subsequently evolves in a Bloch oscillator emulated by a curved array. (**c**) Preparation of two-photon N00N state with arbitrary phase shift *φ*, achieved by detuning the propagation constant by Δ*β* in one of the two arms of a 50:50 directional coupler. (**d**) Radius of curvature *R*_C_ of waveguides arranged in a curved array necessary for reaching a specific point along the Bloch cycle.

We have implemented Bloch arrays of 16 single-mode optical waveguides in glass (Fig. 2b) using the femtosecond laser-writing approach^25^. All the waveguides have identical propagation constants *β*_0_ and are separated by 30 μm such that the coupling coefficients are equal. Here the effective linear ramping potential required to induce BOs is synthetically obtained by bending the waveguides^26^ (see the Methods section).

Two-photon N00N states on a discrete photonic lattice are defined as


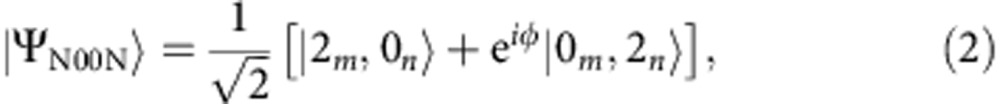


where the indices refer to the lattice sites and *φ* is a relative phase. The quantum correlations in this nonlocal state are such that the two photons always couple into the same excited lattice site, either *m* or *n*, with an equal probability of 1/2. For our experiments, we consider three different two-photon N00N input states in adjacent modes, namely, with symmetric (*φ*=0), antisymmetric (*φ*=*π*) and partially symmetric wave function with *φ*∈(0,*π*). Importantly, all three states are prepared on-chip, which guarantees ultra-high experimental stability. These states are readily achieved by tailoring down-converted light using a detuned directional coupler (DC; Fig. 2c; see the Methods section). Strictly speaking, states generated by type-I spontaneous parametric down-conversion crystals (SPDC) are the so-called two-mode squeezed states^27^ 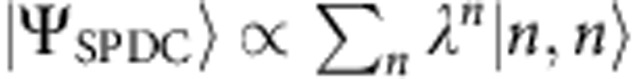. However, in the limit of very weak squeezing and placing ourselves in the coincidence sub-space through trivial post-selection, the source delivers separable, two-photon states of the form 
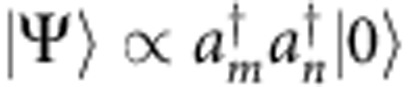
. Then, after mixing through a detuned coupler such states become arbitrary two-photon N00N states (equation (2)).

### Experiments

To monitor the time evolution of quantum correlations, we fabricated several curved arrays of the same physical length (6 cm) but different Bloch periods *λ*_B_. By tuning the radius of curvature (Fig. 2d), we have implemented six arrays for each input state effectively achieving propagation distances of 0.1*λ*_B_, 0.2*λ*_B_, 0.3*λ*_B_, 0.4*λ*_B_, 0.5*λ*_B_ and *λ*_B_ (see the Methods section).

**Figure 3:**
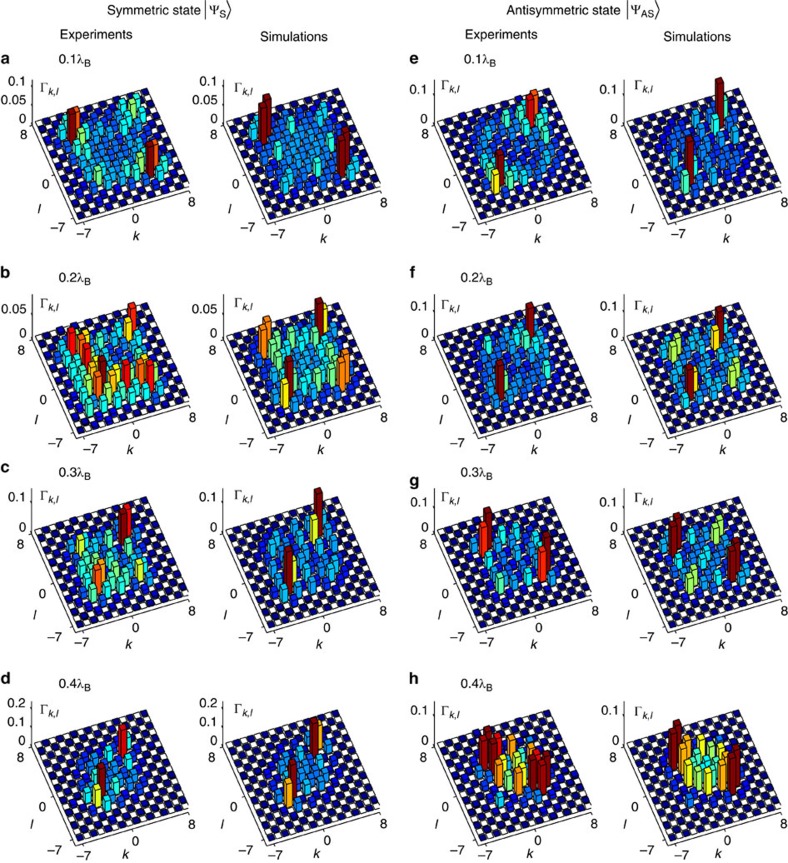
Correlations of two-particle N00N states undergoing Bloch oscillations. Experimental correlation matrices Γ_*k*,*l*_ (*z*) at four propagation distances 0.1*λ*_B_ ... 0.4*λ*_B_ obtained with (**a**–**d**) a symmetric input state 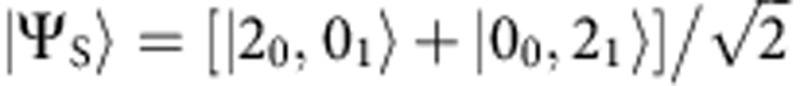 and (**e**–**h**) an antisymmetric input state 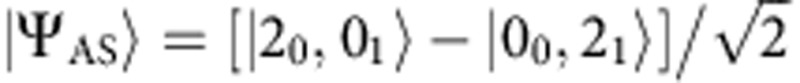. Best-fit corresponding simulations are shown along with experiments, optimized with input phase shifts of 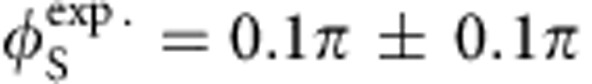 for the symmetric input and with 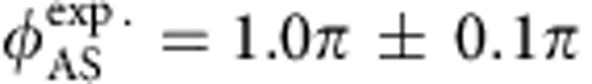 for the antisymmetric one. The coupling coefficient *C* (equal to 0.48 and 0.45 cm^−1^ for symmetric and antisymmetric state preparation, respectively) was also determined through best-fit optimization within a range provided by a coupling-distance-dependence experiment. Non-deterministic number-resolved photon detection was achieved using fibre beam-splitters.

When two indistinguishable particles are present, quantum interference effects can be revealed by analysing twofold correlations. First, we investigate the time evolution of the symmetric state 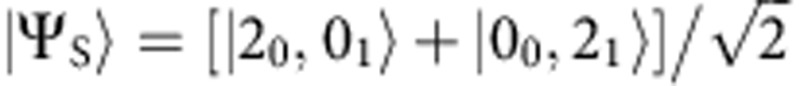, with both photons coupled into either channel zero or channel one of the BO array with the same probability. Once the 
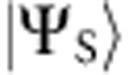
 state is coupled into the system, the spatial correlation function Γ_*k*,*l*_ (*z*) is obtained through measurements of the probability of simultaneously detecting one photon exiting from waveguide *k* and its twin at site *l* (Fig. 2a). Figure 3a–d shows the correlation matrices obtained after the propagation distances of 0.1*λ*_B_, ... 0.4*λ*_B_, respectively. At *z*=0.1*λ*_B_, the correlation map clearly exhibits that the photons initially correlated in their positions have undergone a sudden split-up, becoming anticorrelated (Fig. 3a). Subsequently, after propagating over *z*=0.2*λ*_B_, Γ_*k*,*l*_ (*z*) reveals a correlation turning point with co-occurrence of correlation and anticorrelation among the constitutive photons (Fig. 3b). This regime is a clear manifestation of a fractional symmetry resembling the one expected from anyonic particles–quantum entities that are neither fermions nor bosons ruled by fractional exclusion statistics^24^. After that point the state becomes once more correlated (Fig. 3c). These bunching effects turn out to be evident as the particles approach half of the Bloch cycle (Fig. 3d). Our results are fully supported by best-fit simulations obtained with input phase shift of 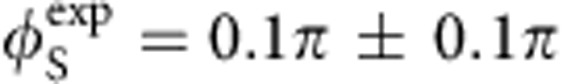 (shown beside in Fig. 3a–d). This small discrepancy when compared with a perfect state can be explained by the presence of losses in the DC section^28^.

**Figure 4:**
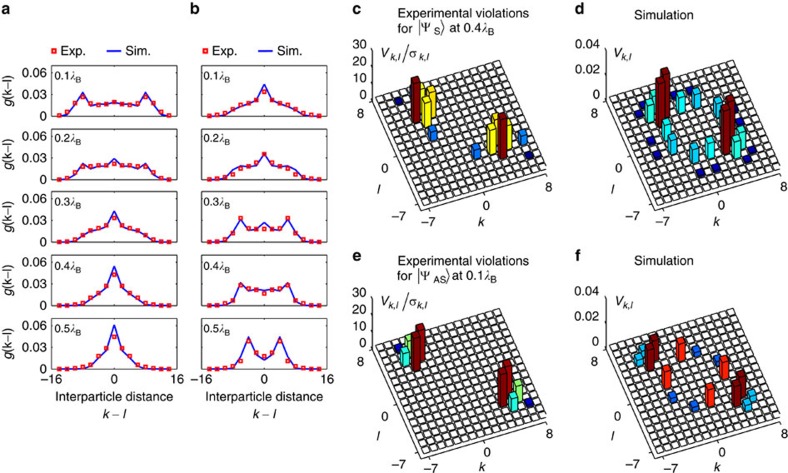
Characterization of bunching to antibunching transitions and observation of nonlocality of the states. (**a**,**b**) Interparticle distance distribution *g*(k−l) at four propagation distances for a symmetric input state 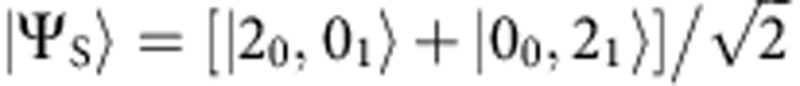 (**a**) and an antisymmetric input state 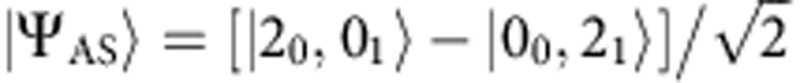 (**b**). (**c**,**d**) Violations of the Bell-like inequality normalized to s.d. obtained with a symmetric input state at a propagation distance of 0.4*λ*_B_, and corresponding simulation. (**e**,**f**) Violations of the Bell-like inequality normalized to s.d. obtained with an antisymmetric input state at a propagation distance of 0.1*λ*_B_ and corresponding simulation. Null or negative elements in the inequality matrix are displayed as a blank cell. The positive elements in the matrix, that is with *V*_*k*,*l*_/*σ*_*k*,*l*_>0, indicate the presence of correlations with no classical analogue.

To gain more insights about the correlation patterns, we extracted the interparticle distance distribution given by 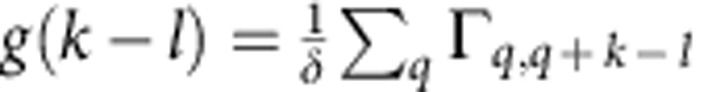. Here *k*−*l* is the distance between sites *k* and *l*, and *δ* is the number of matrix elements Γ_*q*,*q*+*k*−*l*_ satisfying the inequality *q*+*k*−*l*≤*N*. Figure 4a shows how *g* (*k*−*l*) evolves for the symmetric input state. We observe a gradual transition on propagation, from a double-peak shape (antibunching) to a single-peak one (bunching), which confirms that the photon pair passes through a correlation turning point. Furthermore, to evidence probability interference as the underlying mechanism of the antibunching/bunching transition, we performed additional measurements using the device at 0.4*λ*_B_ with two distinguishable photons, which thereby manifest no quantum interference (Supplementary Fig. 1; Supplementary Note 1).

To analyse the impact of the input symmetry property over the photon correlations, as a second case we consider the propagation dynamics of an antisymmetric state, 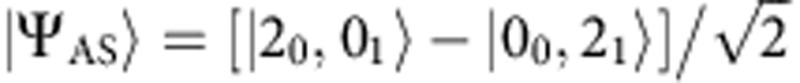. Remarkably, the evolution of this state reveals similar characteristics as observed in the symmetric case (Fig. 3e–h). However, the initial π-phase difference among the modes offsets the correlation cycle to start from a clear bunched state at 0.1*λ*_B_ (Fig. 3e). A singular transition again occurs between the distances 0.2*λ*_B_ and 0.3*λ*_B_, resulting in a strongly anticorrelated state at 0.4*λ*_B_ (Fig. 3h). Extracting the interparticle distance distribution also provides a clear picture of the progressive transformation from correlation to anticorrelation (Fig. 4b). Best-fit simulations are obtained with 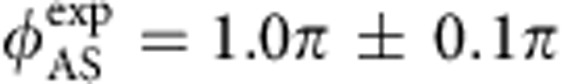 (Fig. 3e–h), and demonstrate the excellent control of the antisymmetric state preparation. For all cases, we measure the correlation similarity between theoretical predictions and our experimental observations, 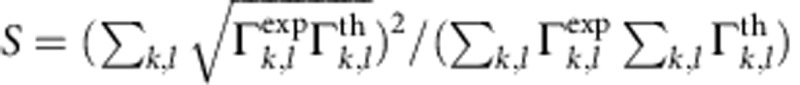. This parameter lies between *S*=0.914±0.001 and *S*=0.952±0.002, which indicates the high performances of our devices (Supplementary Table 1).

To get a whole picture of the influence of the initial state symmetry on the correlation dynamics, as a third case we focus on the evolution of partially symmetric two-photon N00N states. Such photonic states present arbitrary symmetry (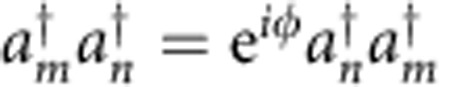, 0<*φ*<*π*) and therefore are able to emulate the behaviour of anyonic particles, which present features of both Bose–Einstein and Fermi-Dirac statistics. For best observation of anyonic-like statistics, we prepared the arbitrary state 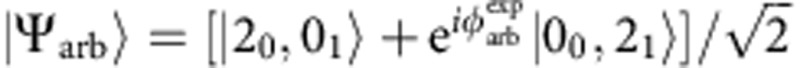, with 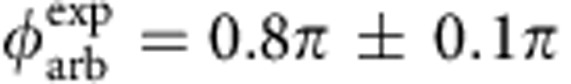. Supplementary Fig. 2 shows the correlation map obtained at fixed propagation distance of 0.3*λ*_B_ along with those obtained for symmetric and antisymmetric state preparation. As anticipated, tuning the input phase shift offsets the correlation cycle. It results in a concurrence of photon bunching and antibunching for the 
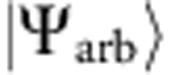
 state input case, and demonstrates the control achieved over the wave function symmetry.

We furthermore estimate the nonclassicality of the states. To do so, the Bell-like inequality 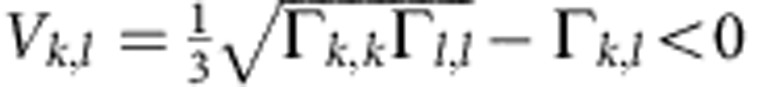 is employed^18^. Its violation provides a valid criterion to discern quantum correlations that manifest themselves as photon bunching. Exemplarily violations for the 
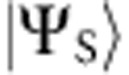
 state occurring at *z*=0.4*λ*_B_ and for the 
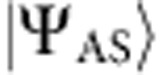
 state at *z*=0.1*λ*_B_ are shown in Fig. 4c,d. The maximum positive value reaches 23*σ*_*k*,*l*_, where the s.d. *σ*_*k*,*l*_ is determined assuming a Poissonian counting statistics. In identical configurations, no violations were observed for distinguishable photons, as expected (Supplementary Note 1).

## Discussion

We have experimentally investigated BOs of two-photon N00N states. For a quantitative assessment of the occurrence of BOs in our experiments, we have performed additional analysis of the data. By extracting the position of one of the particles of the entangled pair in all correlation matrices up to one Bloch period *λ*_B_, the first revival is observed in a heralded measurement with a probability of 0.92±0.02 and 0.94±0.01 for symmetric and antisymmetric state input, respectively (Supplementary Note 2). Beyond the observation of this relocalization effect, which is strictly equivalent to the case of single particles, our experiments reveal for the first time purely quantum features of BOs. In this system, the linear potential continuously induces position-dependent phase shifts on every mode, which has a marked impact on the correlation dynamics. More specifically, quantum states propagating in the lattice experience enhancement or reduction of correlation probabilities depending on the position they are passing through. In particular, this accounts for the presence of correlation turning points, generating oscillations between two diagonal and two off-diagonal peaks in the correlation matrix. Hence, such behaviour can be interpreted as a peculiar form of a spatially extended Hong–Ou–Mandel effect. Furthermore, it reflects the intrinsic capability of the Bloch lattice to efficiently tailor the quantum correlations of the state. The singular transitions evidenced here may be exploited in quantum photonic simulation, as a mechanism to gradually transform a given quantum correlation pattern; for example, for the symmetric input possessing fermionic-like properties at short propagation distances over anyonic- to bosonic-like at half the Bloch cycle. Moreover, we have verified that the correlation cycle is spatially shifted when imposing a phase shift on the input N00N state. More generally, our work sheds light on the unique nonlocal features of two-photon N00N states and demonstrates the high level of control currently obtained with laser-written photonic circuits. In this vein, the generation in a detuned coupler of path-entangled states with fractional symmetry could possibly find applications in topological quantum computing^29^ and in mastering quantum solid-state processes for future computation schemes.

## Methods

### Devices fabrication and design

The waveguides were written inside high-purity fused silica (Corning 7980, ArF grade) using a RegA 9000 seeded by a Mira Ti:Al_2_O_3_ femtosecond laser. Pulses centred at 800 nm with duration of 150 fs were used at a repetition rate of 100 kHz and energy of 450 nJ. The pulses were focused 250 μm under the sample surface using a numerical aperture=0.6 objective while the sample was translated at constant speed of 60 mm min^−1^ (except for the detuned sections) by high-precision positioning stages (ALS130, Aerotech Inc.). The mode field diameters of the guided mode were 18  × 20 μm at 815 nm. At the wavelength of interest, propagation losses and birefringence were estimated at 0.3 dB cm^−1^ and in the order of 10^−7^, respectively. The waveguides are equally spaced by 127 μm at the two facets to match standard V-groove fibre arrays for in- and out-coupling of single photons. The waveguides then smoothly converge through fanning arrangements to their eventual configuration in the functional sections.

### Experimental set-up

Pairs of 815-nm photons with horizontal polarization were produced by type-I optical spontaneous parametric down-conversion from a BiB_3_O_6_ nonlinear crystal pumped with 70 mW from a continuous-wave diode laser, and further filtered by 3-nm interference filters to increase the photon indistinguishability. We successively measured the signals exiting from the even and odd modes, which are collected via a butt-coupled fibre array. They were further coupled with their respective avalanche photodiodes (Fig. 2a) connected to a photon correlator card (Becker & Hickl: DPC230). Data analysis of the channel counts was realized with a time window set at 1 ns. Accidental coincidences, that is, simultaneous detection of two photons not coming from the same pair is estimated to occur with a negligible rate of <2 × 10^−6^ s^−1^. The measurements are acquired over 15 min of integration time and are corrected for coupling fluctuations using simultaneously detected single-photon probability distribution. Each channel is corrected for relative detection efficiencies.

### Arbitrary N00N state preparation

Two-photon N00N states can be created by simultaneously exciting the two modes of an integrated 50:50 DC with indistinguishable photons in a separable product state^30^,^31^. More importantly, the underlying flexibility of this arrangement can be used to conceive a diverse family of two-photon states by adjusting the relative phase *φ* in the range *φ*∈(0,*π*) between the associated propagating modes (Fig. 2c). We accomplish this by inducing an extra-detuning Δ*β* of the propagation constant *β*_0_ in one of the arms of the DC, introducing so-called detuned DCs (Supplementary Methods). During the fabrication process, the control over Δ*β* is readily achieved through adjustment of the writing speed, hence giving access to arbitrary phase shifts.

### On-chip Bloch oscillations

Observing the time dynamics in the BO is made possible by fabricating several devices with different radii of curvature. Indeed, the ramping parameter *B* directly depends on the radius of curvature *R*_C_ of the waveguides according to the expression *B*=Ω/*R*_C_, where Ω is equal to 2*πn*_eff_*d*/*λ*, *n*_eff_ is the effective refractive index of the waveguides, *d* the interwaveguide separation and *λ* the wavelength^26^. As a result, the ramping parameter can be adjusted by tuning *R*_C_, thus affecting the Bloch period. This strategy allows for changes in the effective propagation length while keeping the broadening of the wave function closely comparable between all cases.

## Additional information

**How to cite this article:** Lebugle, M. *et al*. Experimental observation of N00N state Bloch oscillations. *Nat. Commun.* 6:8273 doi: 10.1038/ncomms9273 (2015).

## Supplementary Material

Supplementary Information Supplementary Figures 1-3, Supplementary Table 1, Supplementary Notes 1-2, Supplementary Methods and Supplementary References.

## References

[b1] Bloch F. Über die Quantenmechanik der Elektronen in Kristallgittern. Z. Phys. 52, 555–600 (1929).

[b2] Voisin P. . Observation of the Wannier-Stark quantization in a semiconductor superlattice. Phys. Rev. Lett. 61, 1639–1642 (1988). 10038857 10.1103/PhysRevLett.61.1639

[b3] Longhi S. Optical Bloch oscillations and Zener tunneling with nonclassical light. Phys. Rev. Lett. 101, 193902 (2008). 10.1103/PhysRevLett.101.193902 19113269

[b4] Preiss P. M. . Strongly correlated quantum walks in optical lattices. Science 347, 1229–1233 (2015). 25766229 10.1126/science.1260364

[b5] Morandotti R. . Experimental observation of linear and nonlinear optical Bloch oscillations. Phys. Rev. Lett. 83, 4756–4759 (1999).

[b6] Pertsch T., Dannberg P., Elflein W., Bräuer A. & Lederer F. Optical Bloch oscillations in temperature tuned waveguide arrays. Phys. Rev. Lett. 83, 4752–4755 (1999).

[b7] Dreisow F. . Bloch-Zener oscillations in binary superlattices. Phys. Rev. Lett. 102, 076802 (2009). 19257704 10.1103/PhysRevLett.102.076802

[b8] Bromberg Y., Lahini Y. & Silberberg Y. Bloch oscillations of path-entangled photons. Phys. Rev. Lett. 105, 263604 (2010). 21231662 10.1103/PhysRevLett.105.263604

[b9] Sansoni L. . Two-particle bosonic-fermionic quantum walk via integrated photonics. Phys. Rev. Lett. 108, 010502 (2012). 22304249 10.1103/PhysRevLett.108.010502

[b10] Gräfe M. . On-chip generation of high-order single-photon W-states. Nat. Photonics 8, 791–795 (2014).

[b11] Matthews J. C. F. . Observing fermionic statistics with photons in arbitrary processes. Sci. Rep. 3, 1539 (2013). 23531788 10.1038/srep01539 PMC3609020

[b12] Politi A. . Shor's quantum factoring algorithm on a photonic chip. Science 325, 1221 (2009). 19729649 10.1126/science.1173731

[b13] Heilmann R., Gräfe M., Nolte S. & Szameit A. Arbitrary photonic wave plate operations on chip: realizing Hadamard, Pauli-X, and rotation gates for polarisation qubits. Sci. Rep. 4, 4118 (2014). 24534893 10.1038/srep04118 PMC3927208

[b14] Broome M. A. . Photonic boson sampling in a tunable circuit. Science 339, 794–798 (2013). 23258411 10.1126/science.1231440

[b15] Spring J. B. . Boson sampling on a photonic chip. Science 339, 798–801 (2013). 23258407 10.1126/science.1231692

[b16] Tillman M. . Experimental boson sampling. Nat. Photonics 7, 540–544 (2013).

[b17] Crespi A. . Integrated multimode interferometers with arbitrary designs for photonic boson sampling. Nat. Photonics 7, 545–549 (2013).

[b18] Bromberg Y., Lahini Y., Morandotti R. & Silberberg Y. Quantum and classical correlations in waveguide lattices. Phys. Rev. Lett. 102, 253904 (2009). 19659078 10.1103/PhysRevLett.102.253904

[b19] Peruzzo A. . Quantum walks of correlated photons. Science 329, 1500–1503 (2010). 20847264 10.1126/science.1193515

[b20] Di Giuseppe G. . Einstein-Podolsky-Rosen spatial entanglement in ordered and Anderson photonic lattices. Phys. Rev. Lett. 110, 150503 (2013). 25167236 10.1103/PhysRevLett.110.150503

[b21] Aspuru-Guzik A. & Walther P. Photonic quantum simulators. Nat. Phys. 8, 285–291 (2012).

[b22] Bloch I., Dalibard J. & Nascimbène S. Quantum simulations with ultracold quantum gases. Nat. Phys. 8, 267–276 (2012).

[b23] Blatt R. & Roos C. F. Quantum simulations with trapped ions. Nat. Phys. 8, 277–284 (2012).

[b24] Haldane F. D. M. ‘‘Fractional statistics'' in arbitrary dimensions: a generalization of the Pauli principle. Phys. Rev. Lett. 67, 937–940 (1991). 10045028 10.1103/PhysRevLett.67.937

[b25] Szameit A. & Nolte S. Discrete optics in femtosecond-laser-written photonic structures. J. Phys. B At. Mol. Opt. Phys. 43, 163001 (2010).

[b26] Lenz G., Talanina I. & De Sterke C. M. Bloch oscillations in an array of curved optical waveguides. Phys. Rev. Lett. 83, 963–966 (1999).

[b27] Abouraddy A. F., Nasr M. B., Saleh B. E. A., Sergienko A. V. & Teich M. C. Demonstration of the complementarity of one- and two-photon interference. Phys. Rev. A 63, 063803 (2001).

[b28] Bonneau D. . Quantum interference and manipulation of entanglement in silicon wire waveguide quantum circuits. New J. Phys. 14, 045003 (2012).

[b29] Nayak C., Simon S. H., Stern A., Freedman M. & Das Sarma S. Non-abelian anyons and topological quantum computation. Rev. Mod. Phys. 80, 1083–1159 (2008).

[b30] Hong C. K., Ou Z. Y. & Mandel L. Measurement of subpicosecond time intervals between two photons by interference. Phys. Rev. Lett. 59, 2044–2046 (1987). 10035403 10.1103/PhysRevLett.59.2044

[b31] Marshall G. . Laser written waveguide photonic quantum circuits. Opt. Express 17, 12546–12554 (2009). 19654657 10.1364/oe.17.012546

