# Supplementary material for: Experimental observation of N00N state Bloch oscillations
Source: Nat Commun. 2015 Sep 22;6:8273. doi: 10.1038/ncomms9273 (PMC4595631; doi:10.1038/ncomms9273)
Supplement: Supplementary Information — Supplementary Figures 1-3, Supplementary Table 1, Supplementary Notes 1-2, Supplementary Methods and Supplementary References. [file ncomms9273-s1.pdf]

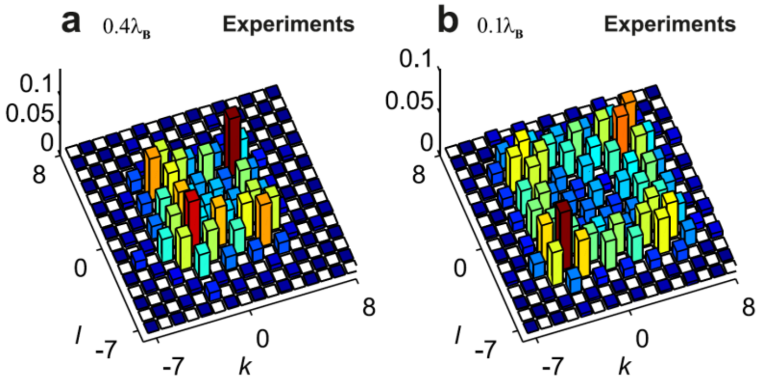

Supplementary Figure 1. Two-photon correlations obtained with distinguishable photons. Experimental correlation matrices $\Gamma_{k,l}(z)$ obtained with pairs of distinguishable photons for **a** the device with symmetric input state preparation at propagation length of $0.4\lambda_{\mathbf{B}}$ and **b** the device with antisymmetric input state at propagation length of $0.1\lambda_{\mathbf{B}}$.

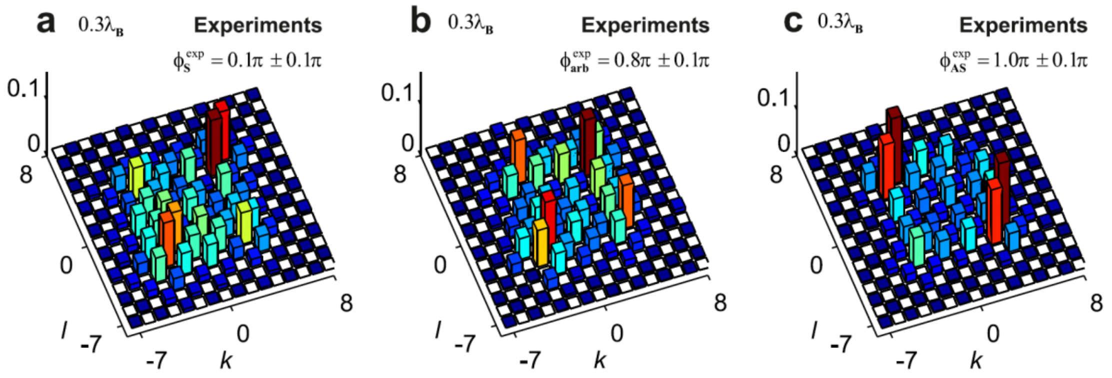

Supplementary Figure 2. Two-photon correlations obtained with different initial phase shifts of the input N00N state. Experimental correlation matrices $\Gamma_{k,l}(z)$ at identical propagation length of $z = 0.3\lambda_{\mathbf{B}}$ obtained through continuous tuning of the input phase shift from **a** to **b** and **c** (see main text). The phase shift of the intermediate case was determined through best-fit optimization procedure between experiments and simulations (not shown).

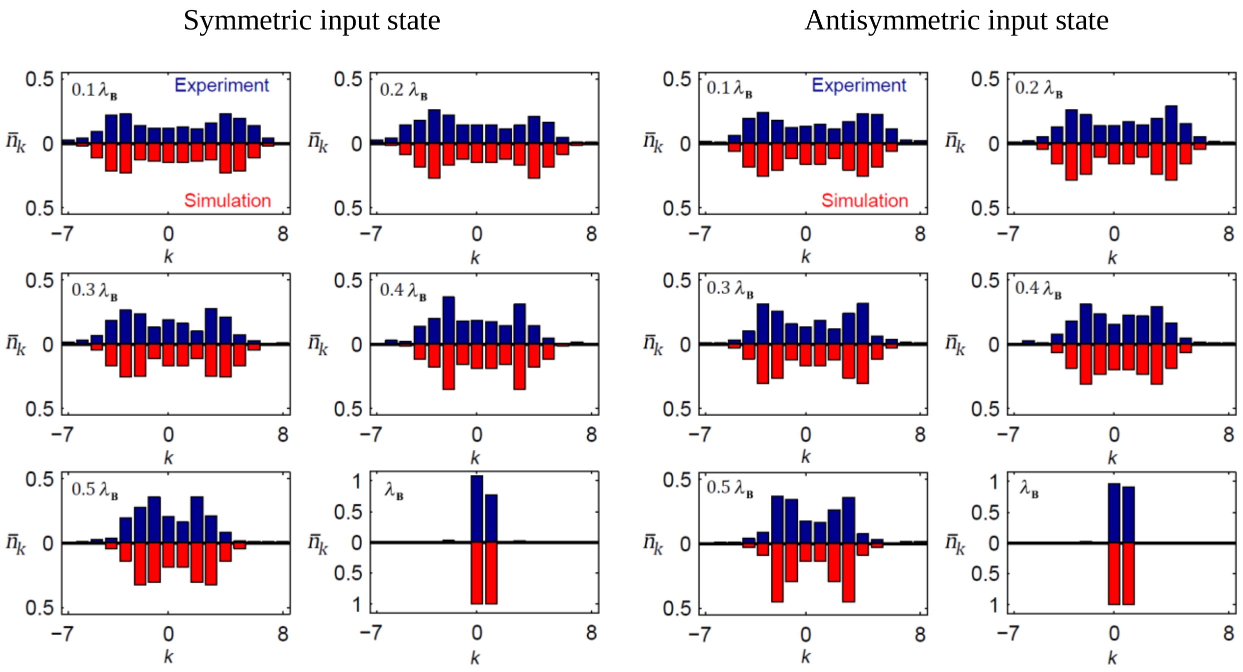

Supplementary Figure 3. Photon densities over a full Bloch cycle. Left: Output experimental photon densities (blue bars) obtained with symmetric input state preparation, with propagation lengths from $0.1\lambda_\mathrm{B}$ to a full Bloch period λ_B. Simulations are shown in red (with reversed vertical axis for better visibility). Right: Output experimental photon densities (blue bars) obtained with antisymmetric input state preparation, with propagation lengths from $0.1\lambda_\mathrm{B}$ to a full Bloch period λ_B. Simulations are shown in red (with reversed vertical axis for better visibility).

Propagation length	$0.1\lambda_\mathrm{B}$	$0.2\lambda_\mathrm{B}$	$0.3\lambda_\mathrm{B}$	$0.4\lambda_\mathrm{B}$
$\lvert\Psi_\mathrm{S}\rangle$ state	0.950 ± 0.004	0.928 ± 0.005	0.924 ± 0.002	0.914 ± 0.001
$\lvert\Psi_\mathrm{AS}\rangle$ state	0.931 ± 0.003	0.952 ± 0.002	0.939 ± 0.006	0.936 ± 0.008

Supplementary Table 1. Similarities between experimental and simulated matrices in the cases of symmetric $\lvert\Psi_\mathrm{S}\rangle$ and antisymmetric $\lvert\Psi_\mathrm{AS}\rangle$ input state preparation. The errors are derived from assuming Poissonian count statistics for and are further propagated for the calculation.

Supplementary Note 1. Matrices with distinguishable particles

We consider the case of the correlations exhibited by the (antisymmetric) symmetric input state at propagation lengths (before) after the turning point of $0.25\lambda_{\mathbf{B}}$ (Fig. 3d and 3e of the main text). These states are strongly correlated, implying that both photons are always found in the same branch, as a result of the cancellation of anticorrelated events. We show here that such amplitude interference is not occurring when deliberately injecting distinguishable photon pairs into the circuit, produced by adding a delay between the two particles that is longer than the wave packet coherence time. Supplementary Figure 1 shows the correlation matrices obtained from injecting such states in the device with symmetric input state preparation with propagation up to $0.4\lambda_{\mathbf{B}}$, and with antisymmetric input state preparation with propagation up to $0.1\lambda_{\mathbf{B}}$. Under these premises, the quantum state remains separable and the correlations map exhibits four peaks. Similar probabilities are therefore observed for the photon pair to gather or separate upon propagation, which is by nature a feature of separable states. Formally, it is a manifestation that time-evolved states of this kind can always be factorized, therefore preventing the building-up of non-classical correlations.

It can be shown that for distinguishable photons, the matrix with elements given by the parameter $V_{k,l} = \frac{1}{3}\sqrt{\Gamma_{k,k}\Gamma_{l,1}} - \Gamma_{k,1} < 0$ is expected to be negative at every position[1]. In our experiments, however, we used indistinguishable photons and, as a result, such matrices exhibit several positive elements, showing a violation of the inequality with an interval of confidence as high as $23\sigma_{k,l}$, where $\sigma_{k,l}$ is the standard deviation.

In order to provide a comparison of the maximum deviation of our experimental non-classical parameters, $V_{k,l}$, with the ones obtained for distinguishable particles; we have determined $V_{k,l}$ normalized to the standard deviation $\sigma_{k,l}$ obtained from the propagation of distinguishable photons in the same Bloch array, that is, $V_{k,l}/\sigma_{k,l}$.

Then, we perform a direct comparison between the matrix elements that maximize the inequality for the indistinguishable case (elements $k,l = 4,-4$ in the violation matrix for the $|\Psi_{\mathrm{S}}\rangle$ state occurring at $z = 0.4\lambda_{\mathbf{B}}$) and the corresponding counterparts with distinguishable photons. By doing so, we obtain a value of -26, that is, the non-classical inequality is not violated with an

interval of confidence as high as $26\sigma_{k,l}$. According to these results, the correlation measurements performed with indistinguishable photons are genuinely quantum.

Supplementary Note 2. Observation of the first Bloch revival

In general, periodicity of a given phenomenon can be assessed by observing its behavior over a full period (e.g. in time or in space). In order to do so, we have determined the photon density $\bar{n}_k(z)$ out of the two-photon correlation measurements covering the propagation distances of $0.1\lambda_{\mathbf{B}}$, $0.2\lambda_{\mathbf{B}}$, $0.3\lambda_{\mathbf{B}}$, $0.4\lambda_{\mathbf{B}}$, $0.5\lambda_{\mathbf{B}}$ and $\lambda_{\mathbf{B}}$ in the Bloch cycle, for both input cases. The heralded photon density was obtained by extracting the marginal $\bar{n}_k = \sum_l \Gamma_{k,l}$ of each correlation matrix, strictly corresponding to the single-photon case. The results are plotted in Supplementary Figure 3, with corresponding simulations. The high quality of the revival of the photon density $\bar{n}_k$ obtained at one Bloch period is evidenced, here estimated to occur with probability of 0.92 ± 0.02 for the symmetric wave function case, and 0.94 ± 0.01 for the antisymmetric one. This indicates the high degree of coherence obtained over an entire Bloch cycle in our experimental realizations.

Supplementary Methods. Detuned directional couplers

To be able to induce a controlled phase shift ϕ of the two-photon N00N state in the range $\phi \in (0, \pi)$, we take advantage of an additional degree of freedom during the fabrication process of a standard directional coupler, namely the writing speed. This parameter controls the amount of deposited laser energy through the number of pulses focused at the same spot in the glass and, therefore, directly varies the mode propagation constant. In doing so, we implement an integrated detuned Directional Coupler (detuned DC). At the single photon level, such detuned DC is described by the coupled set of equations:

$$i\frac{da_0^\dagger}{dz} = \Delta\beta a_0^\dagger - Ca_1^\dagger \tag{1}$$

$$i\frac{da_1^\dagger}{dz} = -Ca_0^\dagger, \tag{2}$$

where $a_k^\dagger$, $(k=0,1)$ is the bosonic creation operator in waveguide k, C is the coupling coefficient and $\Delta\beta$ the difference in the mode propagation constants. After analytical integration of coupled equations (S1) and (S2), we obtain:

$$\begin{bmatrix} a_0^\dagger(z) \\ a_1^\dagger(z) \end{bmatrix} = e^{i\Delta\beta/2} \cdot \begin{bmatrix} \cos(\kappa z) + i\dfrac{\Delta\beta}{2\kappa}\sin(\kappa z) & i\dfrac{C}{\kappa}\sin(\kappa z) \\ i\dfrac{C}{\kappa}\sin(\kappa z) & \cos(\kappa z) - i\dfrac{\Delta\beta}{2\kappa}\sin(\kappa z) \end{bmatrix} \cdot \begin{bmatrix} a_0^\dagger(0) \\ a_1^\dagger(0) \end{bmatrix}, \quad (3)$$

where $\kappa = \sqrt{\left(\frac{\Delta\beta}{2}\right)^2 + C^2}$ is an effective coupling coefficient. A detuned DC behaves as a balanced beam splitter if the condition

$$z = z_{\mathbf{BS}} = \frac{1}{\kappa}\sin^{-1}\left(\frac{\kappa}{\sqrt{2}C}\right) \quad (4)$$

is fulfilled. Remarkably, at the same propagation distance the device is able to transform a two-photon separable product state $a_0^\dagger a_1^\dagger|0\rangle$, where $|0\rangle$ is the vacuum state, into the following state:

$$\begin{aligned} |\Psi(z_{\mathbf{BS}})\rangle &= a_0^\dagger(z_{\mathbf{BS}})\, a_1^\dagger(z_{\mathbf{BS}})|0\rangle \\ &= \exp\left(\frac{2i\Delta\beta\sin^{-1}\left(\frac{\kappa}{\sqrt{2}C}\right)}{\sqrt{4C^2+\Delta\beta^2}}\right)\times \\ &\left[\left(\frac{-\Delta\beta + iC\sqrt{4-\frac{\Delta\beta^2}{C^2}}}{4C}\right)\cdot\left(a_0^\dagger\right)^2 + \left(\frac{+\Delta\beta + iC\sqrt{4-\frac{\Delta\beta^2}{C^2}}}{4C}\right)\cdot\left(a_1^\dagger\right)^2\right]|0\rangle \end{aligned} \quad (5)$$

which is of the form $|\Psi_{N00N}\rangle = \left[|2_m,0_n\rangle + \mathrm{e}^{i\phi}|0_m,2_n\rangle\right]/\sqrt{2}$ up to a global phase factor, and thus identical to equation (2) of the main text. When prepared in such a state, the two photons are always found in the same site with a phase shift between the associated modes given by

$$\phi = \pi - 2\tan^{-1}\sqrt{\left(\frac{2C}{\Delta\beta}\right)^2 - 1}\,. \tag{6}$$

The device therefore allows for on-chip integration of arbitrary path-entangled state presenting mixed quantum statistics, or anyonic-like state. In the particular case of $\Delta\beta = 0$, one can retrieve $\phi = 0$ which corresponds to the well-known situation of a standard DC, where the two photons output the device in a symmetric wave function $|\Psi_{\mathbf{S}}\rangle$. The other specific case is obtained when considering a detuning of $\Delta\beta = 2C$, generating the antisymmetric wave function $|\Psi_{\mathbf{AS}}\rangle$, with $\phi = \pi$. Experimentally, this detuning was achieved by increasing the writing velocity to $80\,\mathrm{mm.min}^{-1}$ in one arm of the coupling section of the detuned DC, compared to $60\,\mathrm{mm.min}^{-1}$ in the other arm. The coupling length was further adjusted according to equation (S4) to obtain again a balanced device, with a splitting ratio of $50:50$. The intermediate situation for generating the arbitrary wave function $|\Psi_{\mathbf{arb}}\rangle$ was obtained with a writing velocity for the detuned mode of $75\,\mathrm{mm.min}^{-1}$ and led experimentally to a phase in the input state of $\phi_{\mathbf{arb}}^{\exp} = 0.8\pi \pm 0.1\pi$.

Every designed detuned DC was first characterized in on-chip interferometers made of one detuned DC followed by a standard DC. The bulk-optic equivalent of this scheme is the detuned Mach-Zehnder interferometer, having two arms of different lengths. Such an arrangement allows for determining the output phase shift of the detuned DC when a classical light beam is launched in one input mode, with appropriate measurement of the output ratio of the interferometer. The designed phase shift was achieved with an accuracy of about $\lambda/10$.

In the second fabrication stage the designed detuned DC was connected to the central modes of the curved waveguide array constitutive of the Bloch-oscillator. After performing the spatial correlation measurements, extensive best-fit optimization of simulated matrices was done through Root Mean Square optimization procedure, with C and ϕ as input parameters. The results are given in the main text, further confirming the introduction of desired phase shifts in the two-photon N00N states.

Supplementary References

1. Bromberg, Y., Lahini, Y., Morandotti, R. & Silberberg, Y. Quantum and classical correlations in waveguide lattices. *Phys. Rev. Lett*. **102**, 253904 (2009).
